# Comprehensive analysis of a glycolysis and cholesterol synthesis-related genes signature for predicting prognosis and immune landscape in osteosarcoma

**DOI:** 10.3389/fimmu.2022.1096009

**Published:** 2022-12-23

**Authors:** Fangxing Xu, Jinglong Yan, Zhibin Peng, Jingsong Liu, Zecheng Li

**Affiliations:** ^1^ Department of Orthopedics, The Second Affiliated Hospital of Harbin Medical University, Harbin, Heilongjiang, China; ^2^ Department of Orthopedics, The First Affiliated Hospital of Harbin Medical University, Harbin, Heilongjiang, China

**Keywords:** glycolysis, cholesterol, osteosarcoma, prognosis, signature, immune, TRAM2

## Abstract

**Background:**

Glycolysis and cholesterol synthesis are crucial in cancer metabolic reprogramming. The aim of this study was to identify a glycolysis and cholesterol synthesis-related genes (GCSRGs) signature for effective prognostic assessments of osteosarcoma patients.

**Methods:**

Gene expression data and clinical information were obtained from GSE21257 and TARGET-OS datasets. Consistent clustering method was used to identify the GCSRGs-related subtypes. Univariate Cox regression and LASSO Cox regression analyses were used to construct the GCSRGs signature. The ssGSEA method was used to analyze the differences in immune cells infiltration. The pRRophetic R package was utilized to assess the drug sensitivity of different groups. Western blotting, cell viability assay, scratch assay and Transwell assay were used to perform cytological validation.

**Results:**

Through bioinformatics analysis, patients diagnosed with osteosarcoma were classified into one of 4 subtypes (quiescent, glycolysis, cholesterol, and mixed subtypes), which differed significantly in terms of prognosis and tumor microenvironment. Weighted gene co-expression network analysis revealed that the modules strongly correlated with glycolysis and cholesterol synthesis were the midnight blue and the yellow modules, respectively. Both univariate and LASSO Cox regression analyses were conducted on screened module genes to identify 5 GCSRGs (RPS28, MCAM, EN1, TRAM2, and VEGFA) constituting a prognostic signature for osteosarcoma patients. The signature was an effective prognostic predictor, independent of clinical characteristics, as verified further via Kaplan-Meier analysis, ROC curve analysis, univariate and multivariate Cox regression analysis. Additionally, GCSRGs signature had strong correlation with drug sensitivity, immune checkpoints and immune cells infiltration. In cytological experiments, we selected TRAM2 as a representative gene to validate the validity of GCSRGs signature, which found that TRAM2 promoted the progression of osteosarcoma cells. Finally, at the pan-cancer level, TRAM2 had been correlated with overall survival, progression free survival, disease specific survival, tumor mutational burden, microsatellite instability, immune checkpoints and immune cells infiltration.

**Conclusion:**

Therefore, we constructed a GCSRGs signature that efficiently predicted osteosarcoma patient prognosis and guided therapy.

## 1 Introduction

Osteosarcoma mostly occurs in the metaphysis of long bone and is the second leading factor of cancer deaths in children and adolescents (1, 2). Currently, surgical resection, chemotherapy, radiation therapy, hormone therapy, and small molecule targeted therapy are the mainstays in osteosarcoma treatment (3). Although the survival rate of osteosarcoma patients has been drastically increased with the combined chemotherapy, the 5-year survival rate is still not ideal for patients with distant metastasis, even with the use of large doses of adjuvant chemotherapy combined with radical resection (4). In addition, the psychological trauma caused by radical resection and the side effects of chemotherapy drugs are also problems that need to be addressed in the current treatment of osteosarcoma. To aid in improving osteosarcoma treatment, identifying novel therapeutic targets and biomarkers is crucial.

Unlike normal cells, cancerous cells often experience metabolic reprogramming. Metabolic reprogramming refers to the modifications to the tumor cells metabolic mode in the starvation state that allow adaption to the nutritional microenvironment; that is, to accommodate the requirements of their own quick growth through sufficient nutrients intake, metabolic reprogramming is a vital hallmark of malignant tumors (5). Glycolysis produces a small amount of energy during the entire glucose metabolism process. Normal cells mainly obtain energy through aerobic respiration. However, cancerous cells deviate from normal cells in various aspects. Even in an aerobic condition, cancerous cells favor the consumption of extra glucose for aerobic glycolysis in order for lactate production, a phenomenon referred to as Warburg effect (6). Calcium-binding protein A10 can accelerate glycolysis by mediating the AKT/mTOR signaling pathway in osteosarcoma, thereby enhancing malignancy of osteosarcoma cells (7). In addition, the novel lncRNA HCG18 enhances aerobic glycolysis in osteosarcoma cells *via* miR-365a-3p/PGK1 signaling pathway regulation, which accelerating the development of osteosarcoma cells (8). HIF-1α oncogene is present in numerous malignancies, including ovarian, breast, and bladder cancers, and can induce the glycolytic pathway in malignant tumors (9–11).

In recent years, the reprogramming of lipid synthesis has been considered to be another significant metabolic abnormality required for tumor growth, in which changes within the cholesterol biosynthetic pathway are vital (12). Cholesterol accumulation within cancerous cells can influence cell proliferation and metastasis, and enhance tumor microenvironmental adaptability, hence reinforcing tumor incidence and progression (13). Studies have demonstrated that several genes involved in cholesterol production are overactive in malignant tissue, such as squalene monooxygenase and the cholesterol biosynthesis rate-limiting enzyme 3-Hydroxy-3-Methylglutaryl Coenzyme A Reductase (HMGCR), which is upregulated within several types of malignancies, comprising glioma and prostate cancer (14, 15). HMGCR overexpression enhances cancer progression and metastasis, while its inhibition can suppress tumors; therefore, HMGCR has been used to treat solid cancers, hematological cancers, and tumors with drug resistance (16–18). In addition, the copy number of the SQLE locus encoding squalene monooxygenase is also increased in a variety of tumors. This copy number increase has been related to pancreatic cancer radiation tolerance and the development of several cancers within breast, prostate and colorectal cancer, or a poor patient prognosis (19, 20). However, similar to gene heterogeneity, tumor cell metabolism is also highly heterogeneous. In other words, no single universal change occurs within cancer metabolism. Tumorous metabolic changes are mainly characterized by changes in lipid and glucose metabolism. Recently, relevant research has discovered that changes in the combined effects on lipid and glucose metabolism have become vital in pancreatic cancer, breast cancer, and skin malignant melanoma (21–23). High-throughput sequencing technologies are developing rapidly, and researchers possess the better understanding of pathogenic genes for various diseases, which is helpful for the discovery of novel biomarkers and pathogenic mechanisms (24). In recent years, differentially expressed genes have been screened through bioinformatics analysis to construct a prognostic signature for predicting osteosarcoma patient prognosis. For example, Zheng et al. constructed a prognostic signature and a nomogram relied on characteristics and clinical variables, which are used to screen out the tumor suppressor gene FHIT in osteosarcoma (25). However, to our knowledge, no gene signature related to glycolysis and cholesterol synthesis has been established to predict osteosarcoma patient prognosis.

During this research, relying on glycolysis and cholesterol synthesis-related genes (GCSRGs), osteosarcoma patients were categorized into one of 4 subtypes, and the differences in patient prognosis and tumor microenvironment between subtypes were also studied. A GCSRGs signature and an efficient nomogram were constructed by screening gene modules and their core genes for associations with glycolysis and cholesterol synthesis. In addition, the relationship of GCSRGs signature with drug sensitivity, immune infiltration and immune checkpoints was investigated, thereby expanding the genes signature’s prognostic values for patients with osteosarcoma. Finally, we performed *in vitro* functional experiments and pan-cancer analysis to validate the genes of interest among the GCSRGs.

## 2 Materials and methods

### 2.1 Data download

GSE21257 dataset (n=53) was downloaded from the Gene Expression Omnibus (GEO) database (https://www.ncbi.nlm.nih.gov/geo/) and the Therapeutically Applicable Research to Generate Effective Treatment-Osteosarcoma (TARGET-OS) dataset (n=95) was obtained from the TARGET database (https://ocg.cancer.gov/programs/target). Both osteosarcoma datasets contain RNA sequences and clinical information. To obtain the total cohort dataset for subsequent mining, we combined TARGET-OS normalized by log2 of the transcript count per million (TPM) and GSE21257 with the batch effect removed by the ComBat function. **Supplementary Table 1** illustrates all patients’ clinical information in the total cohort. GCSRGs were obtained from the “REACTOME_GLYCOLYSIS” (n=72) and “REACTOME_CHOLESTEROL_BIOSYNTHESIS” (n=25) datasets in the Molecular Signatures Database (MSigDB) (https://www.gsea-msigdb.org/gsea/msigdb/). In addition, we downloaded the original pan-cancer mRNA matrix data, clinical data and copy number data from the University of California, Santa Cruz (UCSC) database (https://xenabrowser.net/).

### 2.2 Identification of the GCSRGs-related subtypes

Based on the expression of GCSRGs, the total cohort excluded metabolic genes with a standard deviation ≤ 0.5 and then used the genes as the main objects to perform consistent clustering using ConsensusClusterPlus R package to remove co-expressed metabolic genes and obtain co-expressed GCSRGs. The median expression level classified the metabolic subtypes, which were the quiescent type (glycolysis ≤ 0, cholesterol synthesis ≤ 0), glycolysis type (glycolysis > 0, cholesterol synthesis ≤ 0), cholesterol type (glycolysis > 0, cholesterol synthesis > 0), and mixed type (glycolysis > 0, cholesterol synthesis > 0). The prcomp function was used for principal component analysis (PCA) between subtypes, and survival R package and survminer R package analyzed survival differences between subtypes. The ESTIMATE algorithm calculated tumor purity, immune, stromal, and ESTIMATE scores in different subtypes.

### 2.3 Construction of weighted gene co-expression network and enrichment analysis

Weighted gene co-expression network analysis (WGCNA) employs gene expression data for scale-free network construction. For the top 25% of expression profiles in terms of variation coefficients, we built a network using the WGCNA R package. The modules strongly correlated with glycolysis and cholesterol subtype were screened, and the genes in the modules were pooled as key metabolic genes. Enrichment analysis of GO and KEGG pathway was conducted using clusterProfiler package.

### 2.4 Establishment and validation of a GCSRGs prognostic signature

To screen prognosis-related genes, in a random manner we categorized the total cohort into training and verification cohort, and utilized survival R package to do univariate Cox regression analysis upon the key modules’ genes in training cohort. In order to further minimize the dimensionality and build the risk signature, least absolute shrinkage and selection operator (LASSO) Cox regression analysis has been conducted *via* glmnet R package and survminer R package, and patients’ risk scores were then determined. The training, verification, and total cohorts were categorized into high- and low-risk groups based on risk score’s median value. Survminer R package and survivalROC R package generated survival and receiver operating characteristic (ROC) curves for the high- and low-risk groups. Area under curve (AUC) determined the signature’s predictive ability. Once AUC > 0.6, signature became reliably predictive. We then performed univariate and multivariate Cox regression analyses to see if the risk score was an independent prognostic factor for osteosarcoma patients.

### 2.5 Nomogram construction and validation

The rms R package plotted the clinical nomogram. Performance of nomogram in predicting overall survival (OS) of osteosarcoma patients was evaluated using independent risk factors such as sex, age, metastatic status, and risk score. The calibration curve then proved the nomogram’s efficacy.

### 2.6 Analysis of immune landscape and drug sensitivity

The single-sample gene set enrichment analysis (ssGSEA) method analyzed immune cells infiltration differences across the high- and low-risk groups. Differential expression analysis of immune checkpoints was used to assess the difference in the efficacy of immunotherapy. The pRRophetic R package was utilized to assess the drug sensitivity of different groups.

### 2.7 Pan-cancer analysis of TRAM2

To perform additional research into the role of TRAM2 in tumors, TRAM2 differential expression was assessed in pan-cancer, and we performed a correlation analysis of TRAM2 with patient prognosis, tumor mutational burden (TMB), and microsatellite instability (MSI). Furthermore, we performed a co-expression analysis of TRMA2 with immune cells and immune checkpoints.

### 2.8 Cell culture and transfection

All cell lines had been obtained from Procell (Wuhan, China). These cell lines were cultivated into DMEM/F12 medium containing 10% fetal bovine serum. TRAM2 siRNA and the corresponding si-control had been bought from GenePharma (Shanghai, China). Lipofectamine 3000 reagent (Invitrogen, California, USA) transfected cells as per the guidelines. After 48h of transfection, cells were utilized for protein quantification. The following sequences were utilized for the targeting of TRAM2: 5’-GCGUCCUCAUCGGGCUUAUTT-3’ (si-TRAM2-1); 5’-CCUCGGUGAUUUGGUGCUUTT-3’ (si-TRAM2-2); 5’-GCACGCACUUCCUGAGCUATT-3’ (si-TRAM2-3).

### 2.9 Western blotting

In a nutshell, the protein samples were first isolated using SDS-PAGE. Later, proteins on the gel were moved to PVDF membrane and blocked. Primary antibodies were incubated overnight at a temperature of 4 °C, including anti-TRAM2 (Proteintech, 13311-1-AP, Wuhan, China), anti-E-cadherin (Proteintech, 20874-1-AP), anti-N-cadherin (Proteintech, 22018-1-AP), anti-Vimentin (Proteintech, 10366-1-AP), and anti-GAPDH (Zhongshanjinqiao, TA-08, Beijing, China). On day 2, the membrane underwent secondary antibody incubation. Next, enhanced chemiluminescence (ECL) color developing solution was utilized to develop the membrane after it had been rinsed with TBST three times.

### 2.10 Cell viability assay

The transfected cells have been cultured within 96-well plates at 5000 cells/well. Prior to Detection, Cell Counting Kit 8 (CCK8) reagent (Dojindo, Kumamoto, Japan) was added and incubated at 37 °C. A microplate reader took 450 nm absorbance readings once every 24 h up until 72 h.

In order to evaluate the osteosarcoma cells’ capabilities for colony formation, a plate cloning assay was carried out. The transfected cells were evenly seeded in 6-well plate, and then cultured for 12 days with periodic replacements of the medium. Fixation and staining were accomplished with paraformaldehyde and crystal violet staining solution. A digital camera was used to snap photographs of the cells and recorded data.

### 2.11 Migration and invasion assays

To determine if osteosarcoma cells underwent migratory changes, a scratch assay was performed. 6-well plate was seeded with the transfected cells. After reaching 80% - 90% cell density, the cells were scratched using a pipettor tip oriented perpendicular to the plate’s base. Results were photographed and recorded at 0 h and 48 h.

The invasive potential of osteosarcoma cells was measured using the Transwell assay. After pre-plating the Transwell chamber with Matrigel, the transfected cells were resuspended in fresh basal medium and added to the upper chamber. In the lower chamber, we put in full medium. The upper chamber’s cells were completely removed following 48 h. The remaining cells were stained after fixation, and photographed under a microscope.

### 2.12 Statistical analysis

GraphPad Prism 7 and R (version 3.6.3) were utilized throughout this investigation for all statistical testing and analysis. We used ClusterProfiler R package for consistent clustering. The Kaplan-Meier (KM) method was utilized for the survival analysis, and survival R package performed the log-rank test. In order to conduct LASSO analysis with cross-validation, the glmnet R package was used. The survminer R package and survival R package were used to create the ROC curve. Features selection was performed *via* univariate and multivariate Cox regression analyses. Wilcoxon test compared the continuous variables. Spearman correlation test was used for correlation analysis. P < 0.05 was considered statistically significant unless otherwise stated.

## 3 Results

### 3.1 Identification of the 4 subtypes of osteosarcoma patients by analysis of the expression of GCSRGs

**Figure 1:**
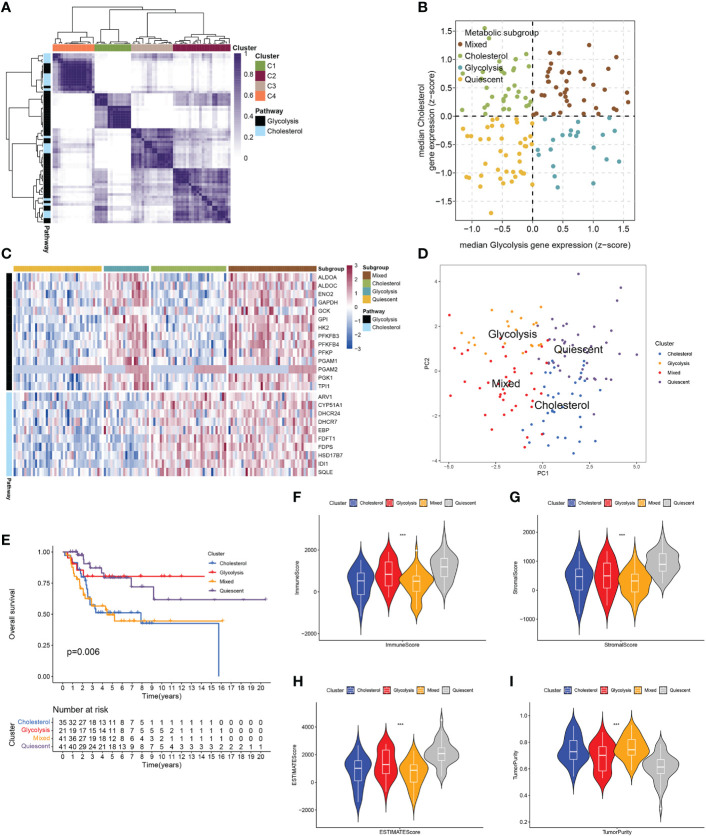
Classification of osteosarcoma patients based on expression of GCSRGs. **(A)** Heatmap showing consensus clustering solution for GCSRGs in osteosarcoma sample **(B)** Scatter plot depicting classification of samples based on GCSRGs expression. **(C)** Heatmap showing expression levels of co-expressed GCSRGs across each subgroup. **(D)** PCA showing significant differentiation between different subgroups of patients. **(E)** Kaplan-Meier survival curves of patients in the different subgroups. Log-rank test P values are displayed. **(F–I)** Violin plots showing the immune score, stromal score, ESTIMATE score and tumor purity across different metabolic subgroups. ***P < 0.001.

The RNA-seq data and clinical information in the GSE21257 dataset and the TARGET-OS dataset were integrated after the batch effect was removed. The total cohort was obtained for subsequent analysis. Based on the gene sets of GCSRGs, metabolic-related genes with a standard deviation ≤0.5 were excluded from the total cohort. Then, consistent clustering was performed using the genes as the main body, thereby removing the co-expressed mixed metabolic genes C2 and C3, and the respective co-expressed metabolic genes were obtained including co-expressed glycolysis genes C1 and co-expressed cholesterol genes C4 (**Figure 1A**). We classified the total cohort into 4 metabolic subtypes based on the median expression levels of GCSRGs. Glycolysis ≤ 0 and cholesterol synthesis ≤ 0 was the quiescent subtype, glycolysis > 0 and cholesterol synthesis ≤ 0 was the glycolysis subtype, glycolysis ≤ 0 and cholesterol synthesis > 0 was the cholesterol subtype, and glycolysis > 0 and cholesterol synthesis > 0 was the mixed subtype (**Figure 1B**). **Figure 1C** illustrates the expression levels of GCSRGs in the 4 subtypes. According to the PCA of the 4 subtypes, the principal components of the 4 subtypes had a good degree of discrimination (**Figure 1D**). Further analysis of the differences in the prognosis between subtypes revealed the significant differences in the prognosis of different subtypes. Among them, prognosis for the glycolysis subtype was significantly better than the cholesterol subtype, and the quiescent subtype’s prognosis was significantly better than the mixed subtype, and the mixed subtype’s prognosis was similar to the cholesterol subtype (**Figure 1E**). In addition, to further analyze the differences in tumor microenvironment between different subtypes, ESTIMATE algorithm ranked the immune, stromal, and ESTIMATE scores as quiescent subtype > glycolysis subtype > cholesterol subtype > mixed subtype, but the reverse trend was noted for the tumor purity (**Figures 1F–I**).

### 3.2 GCSRGs co-expression network and biological activity

**Figure 2:**
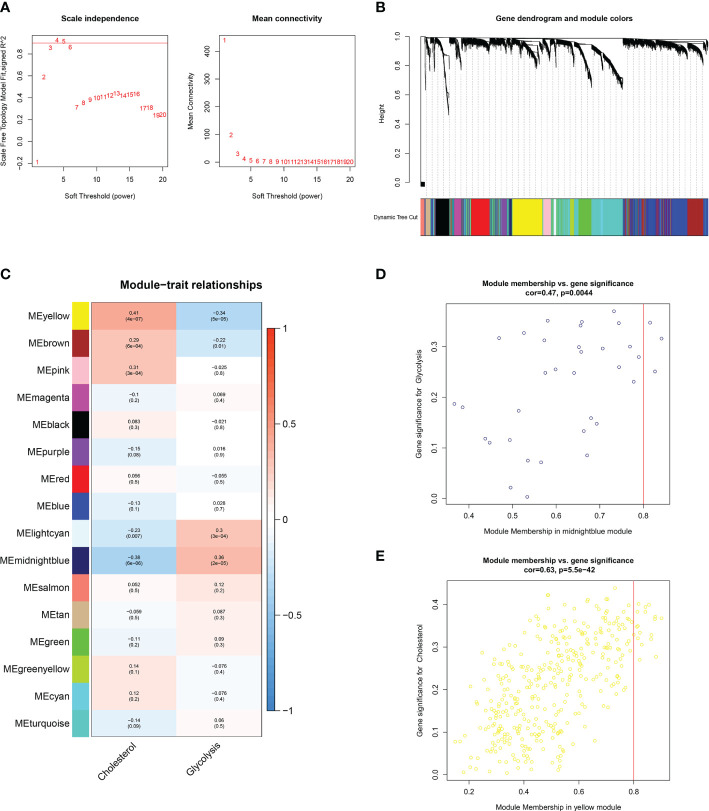
WGCNA to identify similar genes networks of GCSRGs. **(A)** The scale independence (left) and mean connectivity (right) of WGCNA analysis.**(B)** Color coding of co-expression network modules for genes. **(C)** Heatmap showing the correlation of gene modules and glycolysis-cholesterol synthesis. **(D)** Scatter plot displaying the correlation between module membership and gene significance in midnight blue network. **(E)** Scatter plot displaying the correlation between module membership and gene significance in yellow network.

WGCNA was used to discover additional GCSRGs for further studies. The gene network achieved both high internal connectivity and gene similarity when the soft threshold was 4 (**Figure 2A**). Using hybrid dynamic shear tree, with a minimum of 25 genes per gene network module, 16 networks were found to be different from one another and were assigned distinct colors to represent them (**Figure 2B**). Then, the modules with strong correlations with glycolysis and cholesterol synthesis were screened, namely, the midnight blue and the yellow modules (**Figure 2C**). Among them, the glycolysis-related midnight blue module (P = 0.0044) contained 35 genes, and the cholesterol synthesis-related yellow module (P < 0.001) contained 367 genes. **Figures 2D, E** illustrates gene significance and module membership of the 2 modules. A robust positive relationship was identified between these variables’ values.

**Figure 3:**
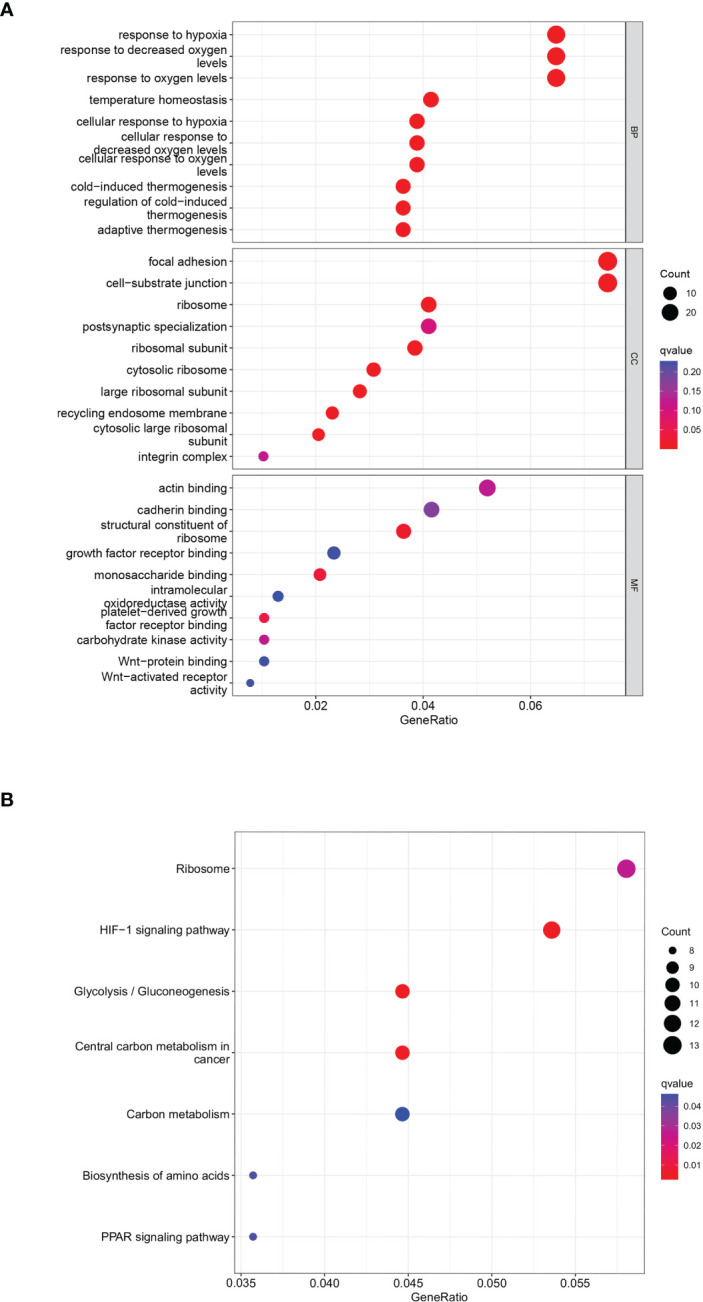
Functional enrichment analysis of genes in the screened modules. **(A)** The results of GO functional enrichment in GCSRGs. **(B)** The results of KEGG pathways enrichment in GCSRGs.

A total of 402 genes within the midnight blue and yellow modules were pooled and used as key metabolic genes. The ClusterProfiler R package was conducted for GO and KEGG pathway enrichment analysis. The bubble plots showed the top 10 in GO-BP, GO-CC, and GO-MF and the top 7 in KEGG. GO functional annotation indicated that GCSRGs were mainly associated with hypoxia response, decreased oxygen response, focal adhesion, cell−substrate junction, ribosome, ribosome structural constituent, and monosaccharide binding (**Figure 3A**). KEGG functional annotation showed that GCSRGs were mainly associated with pathways including ribosome, HIF-1 signaling pathway, glycolysis/gluconeogenesis, and central carbon metabolism in cancer (**Figure 3B**).

### 3.3 Identification and construction of the GCSRGs signature to predict OS in osteosarcoma patients

**Figure 4:**
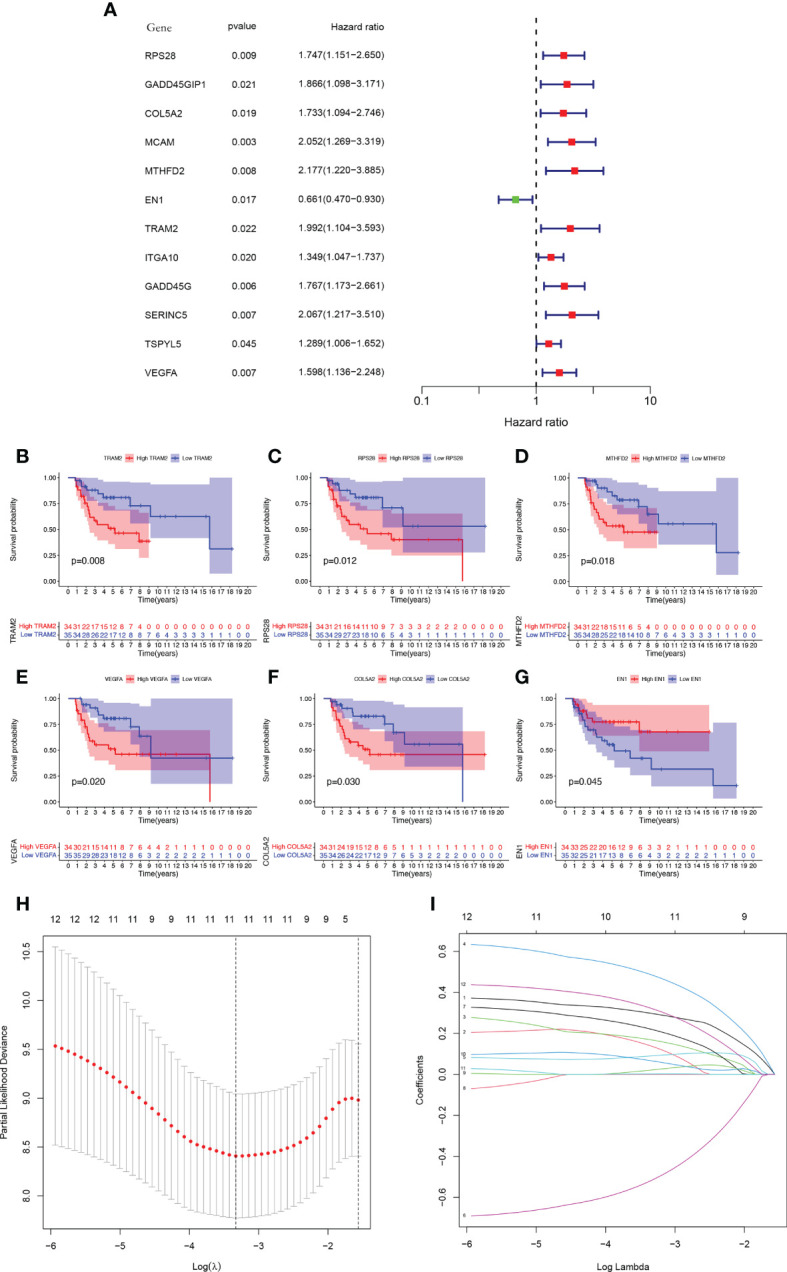
Construction of a GCSRGs prognostic signature in training cohort. **(A)** Forest plot of univariate cox regression analysis of the survival-related 12 differentially expressed genes. **(B–G)** Kaplan-Meier survival curves of patients with differential expression of prognosis-related genes. **(H)** Obtainment of the optimal λ value. **(I)** The LASSO Cox analysis identified 5 genes associated with prognosis.

**Figure 5:**
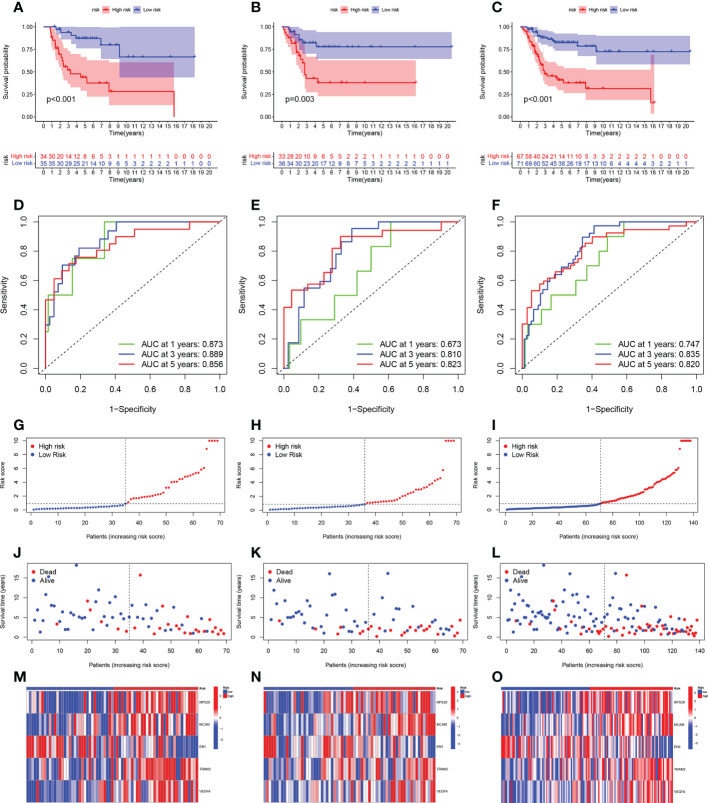
Prognostic value of the GCSRGs signature in training cohort, verification cohort and total cohort. **(A–C)** Kaplan-Meier survival curves according to risk score in the training cohort **(A)**, verification cohort **(B)**, and total cohort **(C)**. **(D–F)** ROC curves for predicting overall survival in the training cohort **(D)**, verification cohort **(E)**, and total cohort **(F)**. **(G–I)** Distribution of risk score in the high-risk group and the low-risk group in the training cohort **(G)**, verification cohort **(H)**, and total cohort **(I–L)** Survival status between the high-risk group and the low-risk group in the training cohort **(J)**, verification cohort **(K)**, and total cohort **(L-O)**.Heatmap of the expression profile of the included glycolysis-cholesterol synthesis related genes in the training cohort **(M)**, verification cohort **(N)**, and total cohort **(O)**.

The total cohort was categorized in a random manner into training and verification cohorts. Univariate Cox analysis was carried out on key metabolic genes (genes in the midnight blue and yellow modules) in the training cohort to screen prognosis-related genes *via* the survival R package, and 12 genes were related to patient prognosis (P < 0.05) (**Figure 4A**). As **Figures 4B–G** indicates, the Kaplan-Meier (KM) survival curves of the top 6 genes from low to high in terms of the P value were listed. Then, LASSO Cox regression analysis further reduced dimensionality and constructed genes signature. In the Cox regression based on the LASSO penalty, as log λ changed, the corresponding coefficient of the determined gene also decreased to 0, and in the cross-validation, 12 genes reached the partial likelihood estimation bias minimum value (**Figures 4H, I**). 5 genes were identified as independent predictors by LASSO Cox regression analysis in training cohort, namely, RPS28, MCAM, EN1, TRAM2, and VEGFA. We determined the risk scores *via* following formula: Risk score = RPS28 × 0.513 + MCAM × 0.701 - EN1 × 0.718 + TRAM2 × 0.575 + VEGFA × 0.467. The training, verification, and total cohorts were all categorized into high- and low-risk groups based on their median risk score. In each of the three cohorts, it was discovered that the low-risk group’s survival probability was significantly greater than the other group (P < 0.005) (**Figures 5A–C**). Then, ROC curve analysis evaluated whether the GCSRGs signature is an efficient prognosis predictor of osteosarcoma patients. The 1-, 3-, and 5-year AUC predicted by the genes signature in training cohort were, 0.873, 0.889, and 0.856, respectively; in verification cohort, were 0.673, 0.810, and 0.823, respectively; in total cohort, were 0.747, 0.835, and 0.820, respectively (**Figures 5D–F**). In the low-risk group, the expression of 4 high-risk genes (RPS28, MCAM, TRAM2, and VEGFA) was low, while the low-risk gene EN1 expression was high (**Figures 5G–I**). Finally, we compared the survival status between the two groups in the three cohorts (**Figures 5J–L**) and plotted an expression heatmap of the risk genes (**Figures 5M–O**).

### 3.4 Independent prognostic analysis of the GCSRGs signature

**Figure 6:**
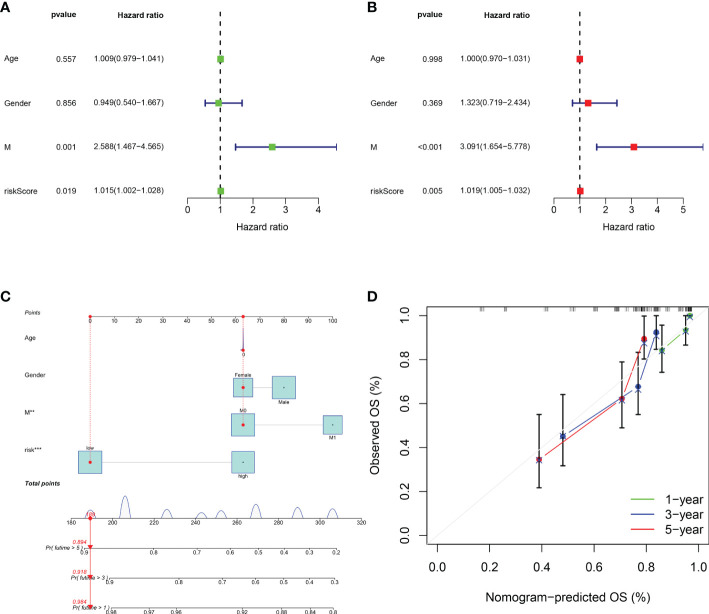
Assessment of the independent prognostic value and construction of the nomogram based on risk score and clinical factors. **(A)** Forest plot of univariate cox regression analysis of various clinical feature and risk score in osteosarcoma. **(B)** Forest plot of multivariate cox regression analysis of various clinical feature and risk score in osteosarcoma. **(C)** The nomogram to predict the 1-, 3- and 5-year survival risk of osteosarcoma patients. **(D)** Calibration curve for the 1-, 3-, and 5-year predicted survival nomogram. **P < 0.01, ***P < 0.001.

To determine if the risk score and the other clinical characteristics are independent prognostic factors for osteosarcoma patients, univariate and multivariate Cox regression analyses were conducted. Univariate Cox regression analysis revealed the risk score (P = 0.019) and the clinical pathological parameters of metastasis (P = 0.001) were independent prognostic factors for osteosarcoma patients (**Figure 6A**), and multivariate Cox regression analysis showed the same results (**Figure 6B**). Furthermore, we developed a prognostic nomogram for estimating the osteosarcoma patients’ survival likelihood (**Figure 6C**). This prognostic nomogram could systematically anticipate the 1-, 3-, and 5-year OS of osteosarcoma patients. The calibration curve showed that actual results were consistent with predicted results (**Figure 6D**).

### 3.5 Immune landscape and drug sensitivity analysis of the GCSRGs signature

**Figure 7:**
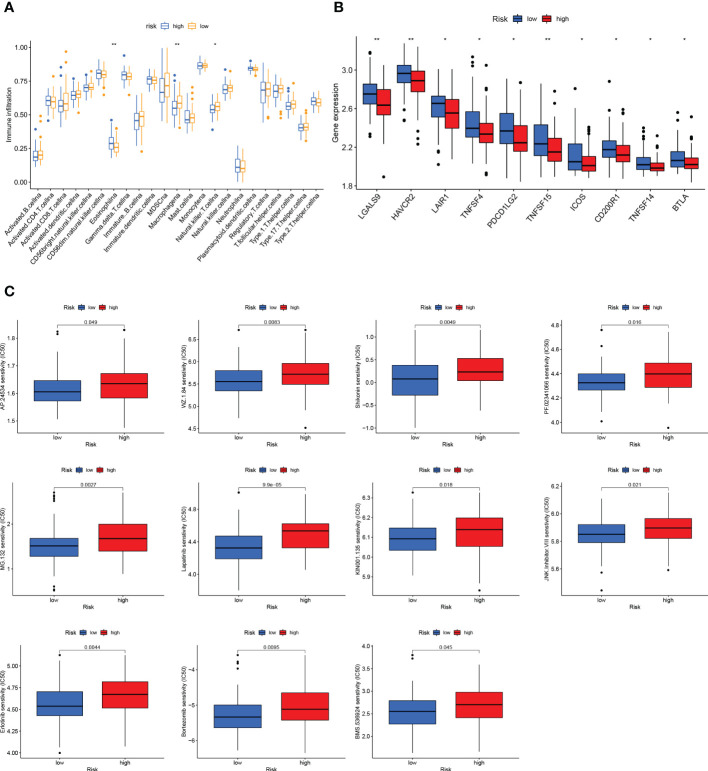
Immune status and drug sensitivity differences between high- and low-risk groups. **(A)** Comparison of immune cell infiltration between the high-risk group and low-risk group. **(B)** Comparison of the expression of immune checkpoints between the high-risk group and low-risk group. **(C)** Drug sensitivity in the high-risk group and low-risk group. *P < 0.05, **P < 0.01.

For confirming if the GCSRGs signature was associated with tumor immunity, we used the ssGSEA method for evaluating differences in immune cells infiltration between the two groups. As **Figure 7A** indicates, the expression of eosinophils, macrophages, and natural killer cells had significant difference between the two groups. Among them, within the high-risk group, eosinophils proportion was significantly increased, while the opposite results occurred in macrophages and natural killer cell proportions. Additionally, as **Figure 7B** indicates, significant differences were found in immune checkpoints expression, including LGALS9, HAVCR2, LAIR1, TNFSF4, PDCD1LG2, TNFSF15, ICOS, CD200R1, TNFSF14, and BTLA between the two groups, with higher expression within the low-risk group than the other, pointing to the fact that there may be limited differences in the efficacy of immunotherapy. Drug sensitivity analysis indicated that 11 drugs were sensitive to patients in the high-risk group (**Figure 7C**), and 13 drugs were sensitive to patients in the low-risk group (**Figure S1**).

### 3.6 Functional verification of TRAM2 *in vitro*

**Figure 8:**
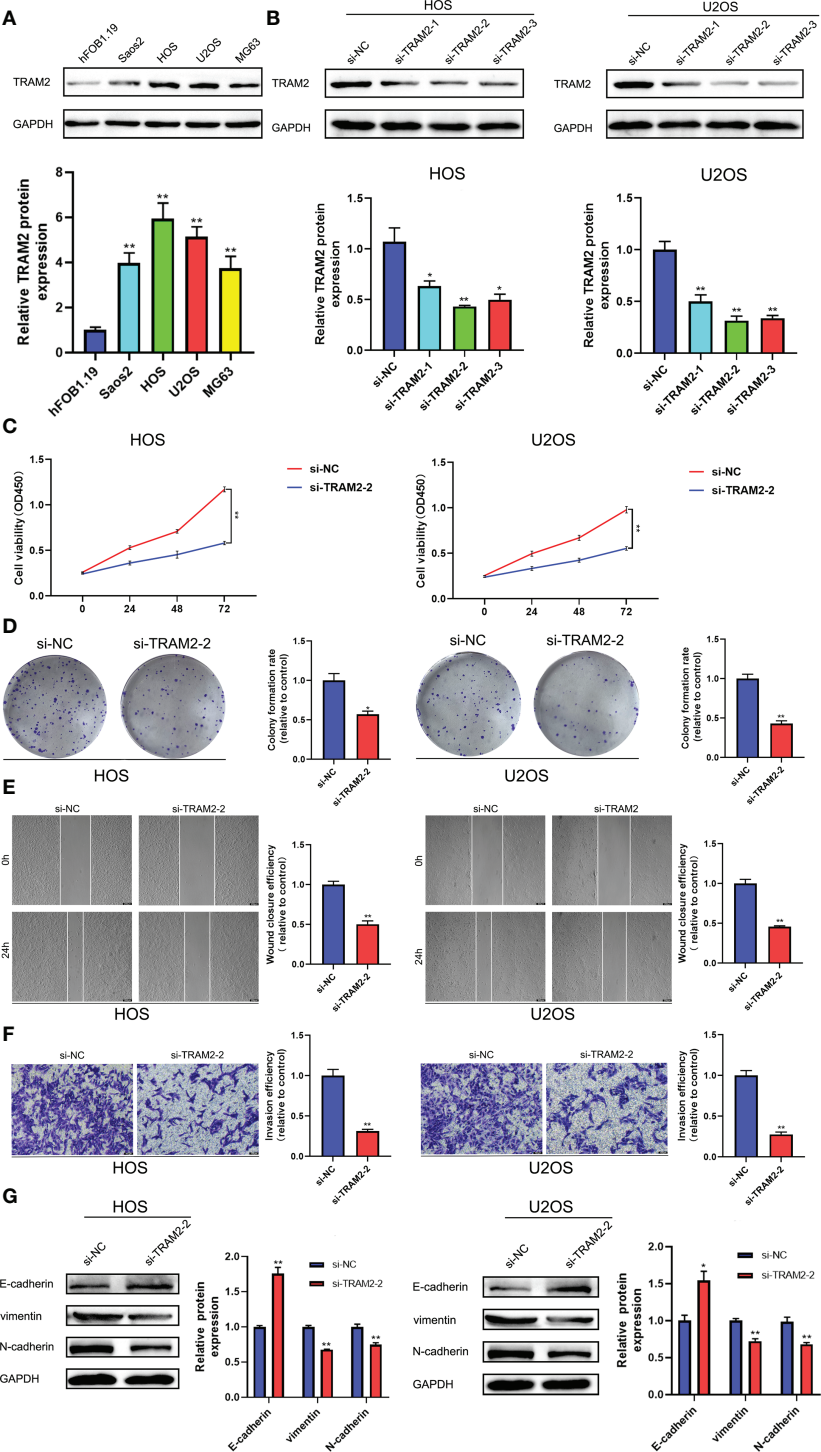
TRAM2 suppressed the progression of osteosarcoma *in vitro*. **(A)** The protein levels of TRAM2 in Saos2, HOS, U2OS, MG63 cells and normal hFOB1.19 cells. **(B)** The protein levels of TRAM2 in HOS and U2OS cells after transfection of si-NC, si-TRAM2-1, si-TRAM2-2 and si-TRAM2-3. **(C)** CCK-8 proliferation assay in HOS and U2OS cells after transfection of si-NC and si-TRAM2-2. **(D)** Plate cloning assay in HOS and U2OS cells after transfection of si-NC and si-TRAM2-2. **(E)** Scratch assay in HOS and U2OS cells after transfection of si-NC and si-TRAM2-2. **(F)** Transwell assay in HOS and U2OS cells after transfection of si-NC and si-TRAM2-2. **(G)** The protein levels of EMT-related proteins including E-cadherin, vimentin and N-cadherin in HOS and U2OS cells after transfection of si-NC and si-TRAM2-2. All results are presented as mean ± SEM. *P < 0.05, **P < 0.01.

We searched the relevant literature of the aforementioned GCSRGs and found that TRAM2 was crucial in some malignancies. However, studies on the mechanism of TRAM2 action in osteosarcoma are scarce. Therefore, TRAM2 is expected to emerge as a promising new biological target in osteosarcoma treatment. Our study first revealed that TRAM2 expression in osteosarcoma cell lines was higher than the human osteoblast cell line according to Western blot results (**Figure 8A**). Then, si-TRAM2 was transferred to HOS and U2OS cell lines to discover the effect of TRAM2 on the osteosarcoma cell progression. Western blot results confirmed transfection efficiency (**Figure 8B**), and si-TRAM2-2 was chosen for further experiments. Based on CCK8 experiment results, TRAM2 downregulation inhibited HOS and U2OS cell lines viability (**Figure 8C**). According to the results of the plate cloning assay, downregulation of TRAM2 expression inhibited the colony-forming ability of the HOS and U2OS cell lines (**Figure 8D**). Furthermore, we conducted cell scratch and Transwell cell invasion assays. Experimental results indicated TRAM2 downregulation inhibited HOS and U2OS cell migration ability (**Figure 8E**) and invasion (**Figure 8F**). Prior studies have revealed that epithelial-mesenchymal transition (EMT) was vital in tumor progression and metastasis (26). So, we examined TRAM2 downregulation effect on EMT-related proteins expression. TRAM2 downregulation promoted E-cadherin expression while suppressing N-cadherin and vimentin expression in the HOS and U2OS cell lines, according to Western blot results (**Figure 8G**).

### 3.7 Pan-cancer analysis of TRAM2

**Figure 9:**
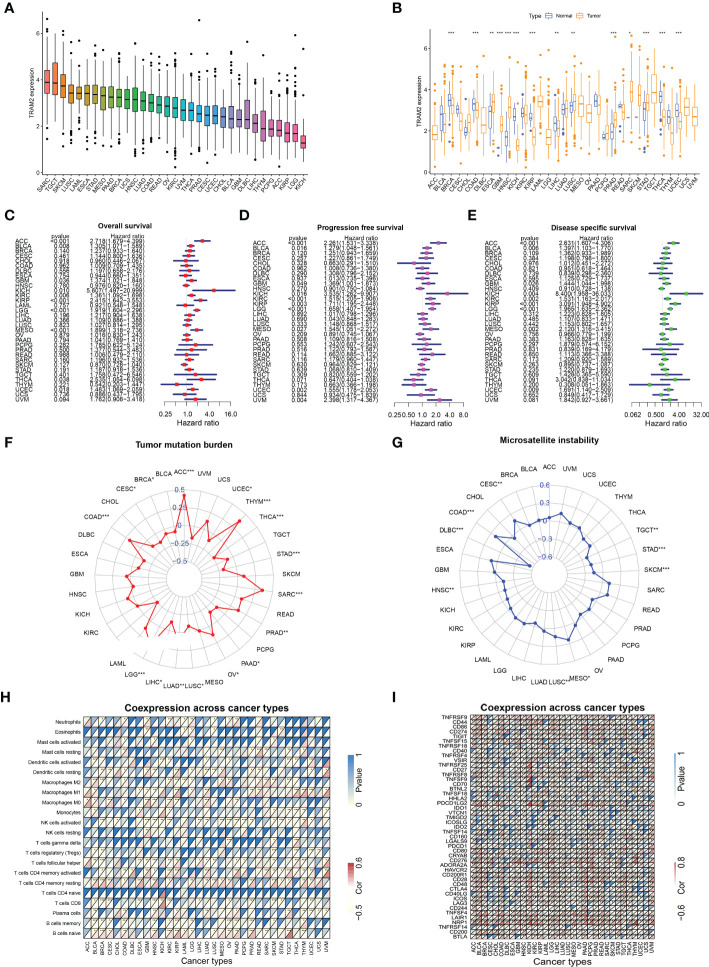
Analysis of TRAM2 in pan-cancer. **(A)** Expression of TRAM2 in 33 cancers. **(B)** Expression of TRAM2 in tumor and normal tissue in pan-cancer. **(C)** Overall survival of TRAM2 in pan-cancer. **(D)** Progression free survival of TRAM2 in pan-cancer. **(E)** Disease specific survival of TRAM2 in pan-cancer. **(F)** Tumor mutation burden of TRAM2 in pan-cancer. **(G)** Microsatellite instability of TRAM2 in pan-cancer. **(H)** Co-expression analysis of TRAM2 and immune cells in pan-cancer. **(I)** Co-expression analysis of TRAM2 and immune checkpoints in pan-cancer. *P < 0.05, **P < 0.01, ***P < 0.001.

To further analyze the important role of TRAM2 in other malignant tumors, we performed pan-cancer analysis of TRAM2. **Figure 9A** shows the expression of TRAM2 in 33 types of cancers, where TRAM2 had the highest expression in SARC. In addition, TRAM2 expression differed significantly between tumor tissues and normal paracancerous tissues in several types of cancer (**Figure 9B**). As shown in **Figures 9C–E**, TRAM2 was relevant to OS, progression free survival (PFS) and disease specific survival (DSS) in a range of cancers. Further analysis of the above data obtained KM survival curves (**Figure S2**). Moreover, TRAM2 was relevant to TMB and MSI in a range of cancers (**Figures 9F, G**). To elucidate the relationship of TRAM2 with immune-related genes and immune checkpoints, we conducted gene co-expression analysis. As **Figures 9H, I** illustrates, TRAM2 can affect immune cell infiltration and immune checkpoint expression in pan-cancer.

## 4 Discussion

Osteosarcoma is a highly invasive cancer. Its poor prognosis is related to problems with current treatments (27). Therefore, there is a need to develop and study prognostic models of osteosarcoma to guide targeted therapy. With the development of bioinformatics and sequencing technology, many scholars have constructed different prognostic models of osteosarcoma to analyze the characteristics of the disease (28–30). However, most of the parameters used to construct prognostic models consider only the genome or transcriptome and do not consider biological processes. As a result, osteosarcoma features cannot be represented accurately within these models. Recently, tumor energy metabolism has attracted increasing interest. Glycolysis and cholesterol synthesis pathways are involved in the metabolic reprogramming of tumors and are crucial in tumor progression (31, 32). In our work, for the first time, we constructed a prognostic signature with glycolysis and cholesterol synthesis as the main characteristics, which can effectively predict osteosarcoma patient prognosis.

We first utilized consensus clustering to confirm the 2 groups of stable independent metabolic genes of glycolysis and cholesterol synthesis and then divided osteosarcoma patients into 4 subtypes (glycolysis subtype, cholesterol subtype, quiescent subtype, and mixed subtype) on basis of median gene expression. Survival across the subtypes showed significant differences based on the prognostic analysis, with the cholesterol subtype and the mixed subtype having the worst prognosis. Additionally, significant differences were observed in tumor purity, scores of immune, stroma, and ESTIMATE, which also confirmed prognosis differences in the 4 subtypes. We used WGCNA to screen out the modules related to glycolysis and cholesterol synthesis and conducted GO and KEGG enrichment analysis. Hypoxia is strongly correlated with poor prognosis, with its pathway activated throughout cancer advancement (33). The HIF-1 protein is heterodimeric with two different subunits, HIF-1α and HIF-1β. This protein activates several genes transcription that encode proteins engaged with angiogenesis, extracellular mesenchymal remodeling, migration, invasion, and metastasis (34). Consistent with the above conclusions, the results of enrichment analysis, such as response to hypoxia and decreased oxygen, and HIF-1 signaling pathway, indicated that this module’s key metabolic genes had tight association with hypoxia process. Previous studies have demonstrated that focal adhesions, as mediators of tumor cells and the extracellular matrix, are vital in various ways within tumor migration, invasion, and drug resistance (35). The results of GO enrichment analysis, such as focal adhesion and cell-substrate junction, indicated that the key metabolic genes in the module may be closely associated with metastasis. Subsequently, univariate Cox and LASSO Cox regression analysis had been conducted on key metabolic genes in the selected modules, and 5 genes (RPS28, MCAM, EN1, TRAM2, and VEGFA) were screened as relevant genes for the GCSRGs signature construction. The GCSRGs signature had good predictive ability in all cohorts and can be utilized as an independent prognostic factor for osteosarcoma patients. Several researchers have investigated the relationship among glycolysis, cholesterol synthesis and immune responses. Regulating cholesterol metabolism can improve CD8 (+) T cells’ anticancer effect (36). Additionally, Li et al. indicated that the glycolysis process of tumor tissues within breast cancer had association with low natural killer T (NKT) cells infiltration (37). In our study, macrophages and NKT cells expression levels within the low-risk group were significantly higher than the other group. According to our knowledge, NKT cells are crucial for controlling tumor cell progression and affecting cancer patient prognosis (38). For macrophages, high infiltration of tumor-associated macrophages in some malignant tumors has a strong correlation with better prognosis (39, 40). That’s consistent with our study findings and helps explain, to a certain extent, why patients who were classified as low-risk group had superior survival outcomes. In addition, our study found that the total 10 immune checkpoint genes expression showed different levels between the two groups, with low-risk group showing higher expression than the other group, indicating that there may be limited differences in the efficacy of immunotherapy.

In our analysis, we selected 5 GCSRGs (RPS28, MCAM, EN1, TRAM2, and VEGFA) as the relevant genes for constructing the risk genes signature. RPS28 is a 40S ribosome component and is critical for 18S rRNA biosynthesis (41). There are few studies on the effect of RPS28 on cancer, and most research results are only predictions generated by bioinformatics and have not been confirmed by corresponding biological experiments (42, 43). However, some researchers have found that reducing the expression of RPS28 protein can reduce the cell viability of HeLa cells and induce tumor cell apoptosis (44), indicating that RPS28 has a major regulatory function in cancer. Additionally, RPS28 can influence tumor immunosurveillance and regulate T cell killing (45). MCAM is highly expressed in various malignancies and has tight association with their growth and metastasis, such as melanoma (46), prostate cancer (47), gastric cancer (48), and lung cancer (49). Prior investigations revealed that MCAM was associated with poor prognosis of osteosarcoma patients and can improve the migration ability of osteosarcoma cells (50). For immunotherapy, MCAM deficiency significantly impairs T cell-mediated antitumor effect (51). Solid tumor progression and metastasis are accompanied by angiogenesis stimulation, with VEGFA as the main factor driving tumor vascular bed expansion (52). VEGFA is involved in angiogenesis, progression, and metastasis in various malignancies, including osteosarcoma, and has a strong association with a poor prognosis (53–55). Moreover, the expression of co-inhibitory receptor and regulatory T cell expansion are both influence by VEGFA signaling (56). Hence, targeted VEGFA therapy is a key area for improving the osteosarcoma prognosis (57). TRAM2 is a translocon component and can transport proteins synthesized by ribosomes to the endoplasmic reticulum (ER), acting as ER channels for calcium concentration regulation within it (58). In glioma, through its PI3K/AKT/mTOR signaling pathway regulation, TRAM2 is able to enhance tumor cells migration, invasion, proliferation, and EMT (59). In addition, TRAM2 and YAP activity in various cancers shows a very strong expression correlation, demonstrating that TRAM2 acts a significant role in malignant proliferation and invasion caused by YAP (60). However, no relevant studies have shown the relationship between TRAM2 and osteosarcoma. Therefore, to ensure the validity of the GCSRGs signature, we chose to use TRAM2 for cell function validation and pan-cancer analysis.

TRAM2 protein expression was demonstrated to be significantly different across the osteosarcoma and the human osteoblast cell lines during experimental validation. In addition, inhibiting of EMT-related protein expression, cell viability, colony formation, migration, and invasion were achieved by downregulating TRAM2 protein expression in osteosarcoma cells. These findings provide further support for validity of genes signature based on glycolysis and cholesterol synthesis and suggest that TRAM2 is involved in osteosarcoma cells progression. In addition, TRAM2 was not only involved in osteosarcoma progression but also closely related to OS, PFS, DSS, TMD, MSI, immune cell infiltration and immune checkpoints in pan-cancer, suggesting that the GCSRGs signature and the target genes in the signature have the potential to serve as the prognostic indicators for a wide range of cancers.

Although we confirmed the effective role of the GCSRGs signature in predicting the prognosis of osteosarcoma patients and confirmed the tumor-promoting effect of TRAM2 in osteosarcoma cells in cytological experiments *in vitro*, this study still has certain drawbacks that require further research. First, the patient sample size was small within the datasets used, and their clinical characteristics were not sufficiently detailed. Therefore, a larger sample size with more detailed clinical characteristics is needed. In addition, besides TRAM2, other signature-related genes should also be verified at the cytological level.

During this research, osteosarcoma patients were categorized into 4 subtypes according to GCSRGs expression matrix, and these subtypes differed significantly from one another in terms of prognosis and tumor microenvironment. Through WGCNA, the gene modules most closely associated with glycolysis and cholesterol synthesis were screened, and a risk signature of osteosarcoma consisting of 5 GCSRGs was constructed for the first time. In addition, we found that this signature was closely related to immune cells infiltration and immune checkpoint expression in osteosarcoma patients. These findings not only provide a new method to predict the prognosis of osteosarcoma patients but also provide novel therapeutic targets.

## Data availability statement

The original contributions presented in the study are included in the article/**Supplementary Material**. Further inquiries can be directed to the corresponding author.

## Author contributions

JY and FX designed the research and drafted the manuscript. FX and ZP completed the bioinformatics analysis. JL and ZL completed cytology experiments. The final manuscript has been read by all of the authors, and they have all given their approval for it to be submitted.

## Supplementary material

Supplementary Figure 1 The 13 drugs were selected for osteosarcoma patients with the low-risk group

Supplementary Figure 2 Correlation between the expression of TRAM2 and survival prognosis in pan-cancer **(A)** Kaplan-Meier survival curves of the relationship between TRAM2 expression and overall survival in pan-cancer. **(B)** Kaplan-Meier survival curves of the relationship between TRAM2 expression and progression free survival in pan-cancer. **(C)** Kaplan-Meier survival curves of the relationship between TRAM2 expression and Disease specific survival in pan-cancer.
