# Supplementary figures and images for: Comprehensive analysis of a glycolysis and cholesterol synthesis-related genes signature for predicting prognosis and immune landscape in osteosarcoma
Source: Front Immunol. 2022 Dec 23;13:1096009. doi: 10.3389/fimmu.2022.1096009 (PMC9822727; doi:10.3389/fimmu.2022.1096009)

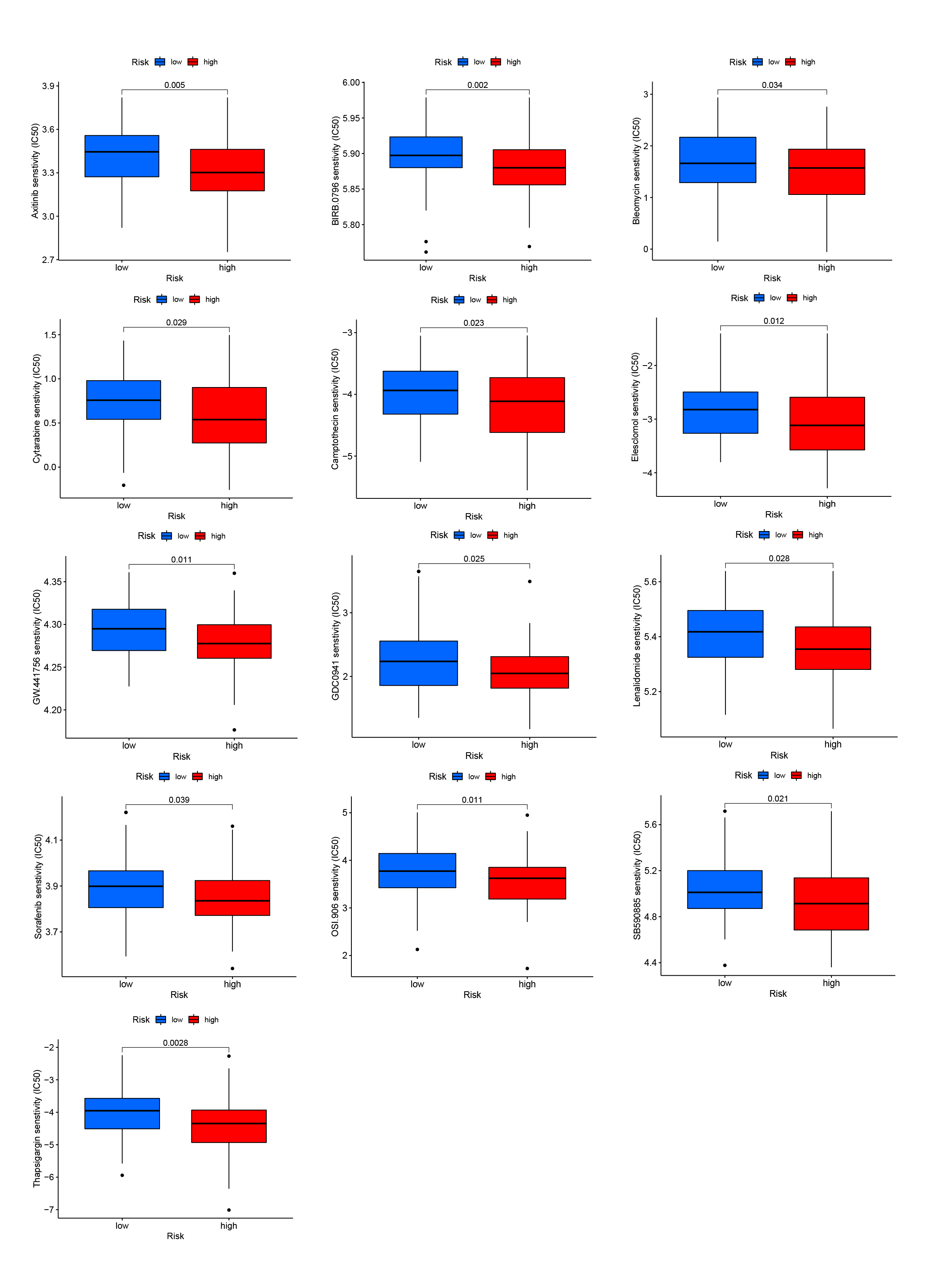

Supplement: Supplementary Figure 1 — The 13 drugs were selected for osteosarcoma patients with the low-risk group [file Image_1.tif]

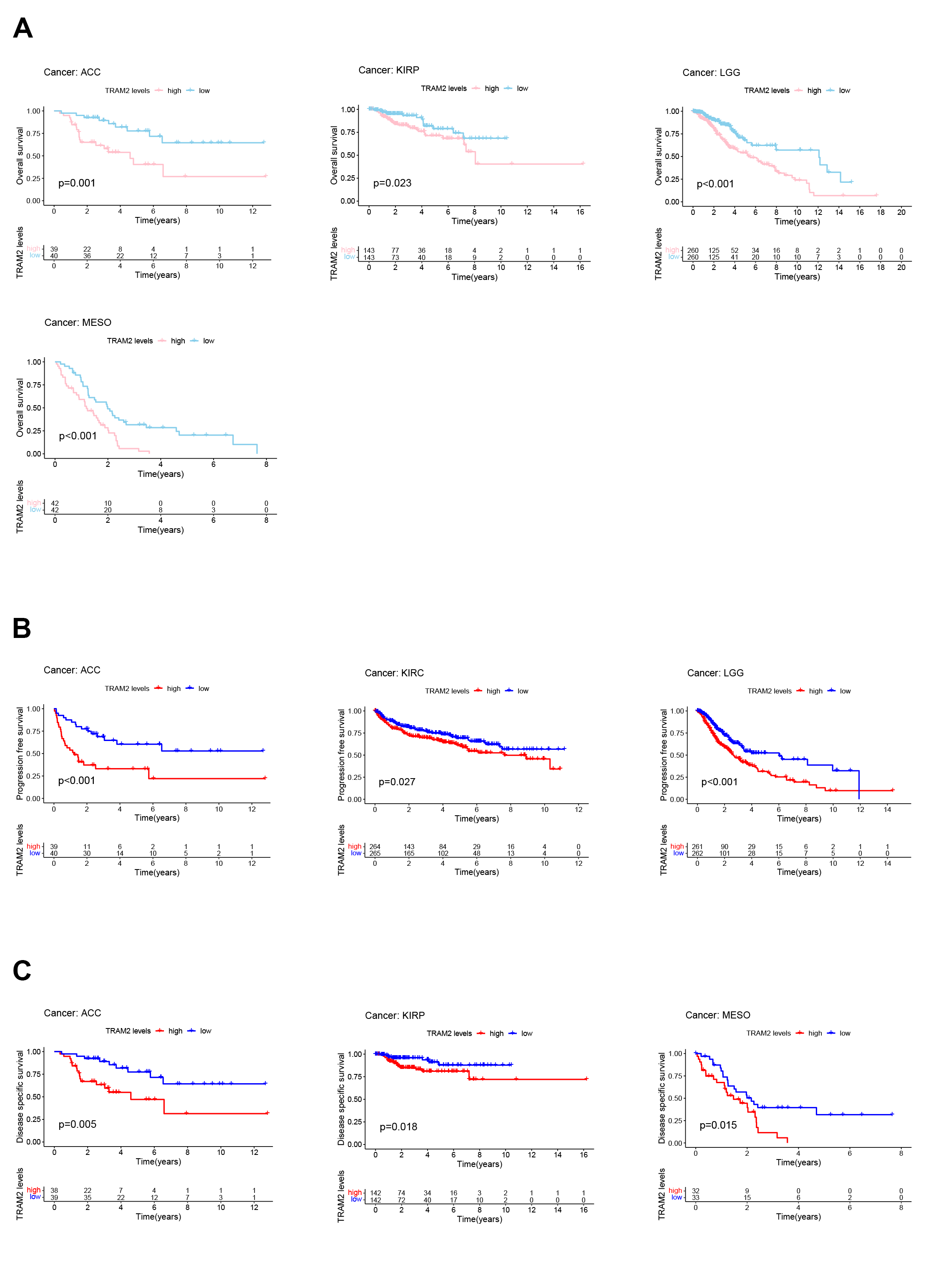

Supplement: Supplementary Figure 2 — Correlation between the expression of TRAM2 and survival prognosis in pan-cancer (A) Kaplan-Meier survival curves of the relationship between TRAM2 expression and overall survival in pan-cancer. (B) Kaplan-Meier survival curves of the relationship between TRAM2 expression and progression free survival in pan-cancer. (C) Kaplan-Meier survival curves of the relationship between TRAM2 expression and Disease specific survival in pan-cancer. [file Image_2.tif]
